# Prognostic Analysis of Limited Resection Versus Lobectomy in Stage IA Small Cell Lung Cancer Patients Based on the Surveillance, Epidemiology, and End Results Registry Database

**DOI:** 10.3389/fgene.2018.00568

**Published:** 2018-11-22

**Authors:** Chang Gu, Zhenyu Huang, Chenyang Dai, Yiting Wang, Yijiu Ren, Yunlang She, Hang Su, Chang Chen

**Affiliations:** ^1^Department of Thoracic Surgery, Shanghai Pulmonary Hospital, Tongji University School of Medicine, Shanghai, China; ^2^Department of Colorectal and Anal Surgery, Xinhua Hospital, Shanghai Jiao Tong University School of Medicine, Shanghai, China; ^3^Department of Radiation Oncology, Shanghai Chest Hospital, Shanghai Jiao Tong University, Shanghai, China

**Keywords:** lung cancer, small cell lung cancer, prognosis, sublober resection, lobectomy

## Abstract

**Objective:** The prognostic analysis of limited resection vs. lobectomy in stage IA small cell lung cancer (SCLC) remains scarce.

**Methods:** Using the Surveillance, Epidemiology, and End Results registry (SEER) database, we identified patients who were diagnosed with pathological stage IA (T1a/bN0M0) SCLC from 2004 to 2013. The overall survival (OS) and lung cancer-specific survival (LCSS) rates of patients with different treatment schemes were compared in stratification analyses. Univariable and multivariable analyses were also performed to identify the significant predictors of OS and LCSS.

**Results:** In total, we extracted 491 pathological stage IA SCLC patients, 106 (21.6%) of whom received lobectomy, 70 (14.3%) received sublobar resection and 315 (64.1%) received non-surgical treatment, respectively. There were significant differences among the groups based on different treatment schemes in OS (log-rank *p* < 0.0001) and LCSS (log-rank *p* < 0.0001). Furthermore, in subgroup analyses, we did not identify any differences between sublober resection group and lobectomy group in OS (log-rank *p* = 0.14) or LCSS (log-rank *p* = 0.4565). Patients with four or more lymph node dissection had better prognosis. Multivariable analyses revealed age, laterality, tumor location, and N number were still significant predictors of OS, whereas age, tumor location, and N number were significant predictors of LCSS.

**Conclusion:** Surgery is an important component of multidisciplinary treatment for stage IA SCLC patients and sublober resection is not inferior to lobectomy for the specific patients.

## Introduction

Lung cancer is the second most commonly diagnosed cancer and the leading cause of death from cancer worldwide (Jemal et al., 2011). Small cell lung cancer (SCLC), as the most common neuroendocrine tumor, comprises almost 14% of all lung cancer patients (Siegel et al., 2016). Besides, SCLC is recognized as an aggressive neoplasm characterized by rapid growth and early development of widespread metastases (especially hematogenous metastases) (Ettinger et al., 2018). When compared with non-small cell lung cancer (NSCLC), the 5-year overall survival rate is only 6.2% while the rate reaches to 18.0% in NSCLC patients (Kalemkerian et al., 2013).

As the Veterans Administration (VA) Lung Study Group proposed, SCLC is typically classified as limited-stage and extensive-stage disease (Argiris and Murren, 2001). Approximately 30% of SCLC patients present with limited-stage disease, most of whom have experienced lymphatic metastasis at their first diagnosed. SCLC is sensitive to chemotherapy and radiotherapy. Thus, systemic therapy is recommended for all patients with SCLC by the National Comprehensive Cancer Network (NCCN) Guidelines. However, the NCCN Guidelines indicates that, for stage I SCLC patients without mediastinal lymph node metastasis, surgery should be considered (Ettinger et al., 2018).

In early-staged NSCLC patients, surgical resection could offer a potential cure in clinical practice (Islam et al., 2013). Lobectomy with mediastinal lymph mode dissection has been recommended as the standard scheme for early-staged NSCLC patients (Darling et al., 2011). However, limited resection (anatomical segmentectomy and non-anatomical wedge resection) is considered as a compromising surgical procedure for high-risk patients, whereas it has the advantage of preserving lung function and providing the chance for a second operation (Kocaturk et al., 2011; Zuin et al., 2013; Gu et al., 2017). Although the efficacy of limited resection for early-staged NSCLC patients has been doubted, many studies have proved it achieves equivalent oncological outcomes to lobectomy, no matter in the elderly or the young set (Altorki et al., 2014; Sihoe and Van Schil, 2014; Gu et al., 2017).

As for early-staged SCLC patients, the role of surgery has not been assessed by any prospective studies. However, data from retrospective studies showed favorable results when additional surgery was applied in patients with stage I SCLC (James et al., 2010; Yang et al., 2016). And the 5-year survival rate could be improved to 40–60% by surgery in stage I SCLC patients (James et al., 2010). However, given the characteristics of rapid growth and the sensitivity to chemoradiotherapy, limited resection, especially for high-risk patients, is only recognized as a compromise procedure by many surgeons. Few studies evaluated the oncological effect of limited resection and the equivalency of limited resection verse lobectomy among stage IA SCLC patients. In this study, we used the population-based Surveillance, Epidemiology, and End Results (SEER) registry to compare the oncological efficacy between limited resection and lobectomy in patients with stage IA SCLC patients, and further investigated the prognostic factors for these patients.

## Materials and Methods

### Study Population

The study population was confined to patients who were diagnosed with pathological stage IA (T1a/bN0M0) SCLC from 2004 to 2013 in SEER database. The exclusion criteria were as follows: (1) patients with a second primary neoplasm or with synchronous multiple primary lung cancer; (2) surgical patients treated with neoadjuvant/intraoperative radiotherapy, which could be neoplasms of higher stage; (3) patients with lung metastases (pathologically conformed SCLC) from other locations; (4) unknown tumor location or primary main bronchus tumor; (5) patients with unknown medical records on survival status. All the data extracted from the Surveillance, Epidemiology, and End Results registry, which is a public population-based database and the Institutional Review Board of our hospital approved our study with a waiver for the requirement of patient consent.

The codes of tumor histology were consistent with the International Classification of Diseases for Oncology (Percy et al., 1990). Relevant sociodemographic information was extracted from SEER database, along with all the available tumor features, including age, gender, race, laterality (left or right), primary tumor location (which lobe), grade, tumor size, and treatment strategy. Tumor pathologic TNM stage were determined according to the 7th edition of TNM staging system proposed by the American Joint Committee on Cancer (AJCC) (Edge et al., 2010).

Survival time was defined as the time frame of the date of diagnosis to the date of death. Patients still alive at the time point of December 31, 2013 were set as censored cases. Furthermore, deaths from other causes were censored at the time of death when investigating the lung cancer specific survival (LCSS).

### Statistical Analysis

All the patients were grouped by treatment strategies and the baseline variables of different groups were compared. Data with continuous covariates were presented as median ± standard deviation (SD) and were analyzed using Student’s *t*-test while data with categorical covariates were presented as number (%) and were analyzed using Pearson χ^2^ test. The distributions of overall survival (OS) and LCSS were calculated with Kaplan-Meier method, and the significance among different groups was explored by the log-rank test. Furthermore, a Cox proportional hazards model was established to probe prognostic factors for OS and LCSS by univariable and multivariable analyses.

All the clinicopathological data were analyzed using SPSS 22.0 software package (SPSS Inc., Chicago, IL, United States) while the distributions of OS and LCSS were draw utilizing Prism 5 (Graph Pad Software Inc., La Jolla, CA, United States). Statistical significance was set as *p* < 0.05.

## Results

Totally, we identified 491 stage IA SCLC patients from SEER database. There were 106 (21.6%) patients received lobectomy, 70 (14.3%) received sublobar resection, 215 (43.8%) received adjuvant radiotherapy alone, and 100 (20.3%) received no treatment, respectively. Furthermore, of all the patients who underwent surgical resection, 83 patients underwent lobectomy only, 23 underwent lobectomy plus adjuvant radiotherapy, 54 underwent sublober resection only, and 16 underwent sublober resection plus adjuvant radiotherapy, respectively.

The baseline characteristics of all the patients were listed in Table 1. The elderly patients account for the majority of the patient sets. Based on the data in Table 1, there was no statistical difference among the three groups of different treatment schemes in gender, race and tumor location. However, compared with patients who had surgical treatment, patients without surgical resection were apt to had older age (*p* < 0.001), higher tumor stage (*p* = 0.003), larger tumor size (*p* < 0.001), and more radiotherapy (*p* < 0.001). Moreover, between patients underwent sublobar resection and patients received lobectomy, there was no significant difference in age at diagnosis (*p* = 0.152), gender (*p* = 0.994), race (*p* = 0.464), laterality (*p* = 0.129), tumor location (*p* = 0.071), T stage (*p* = 0.275), tumor size (*p* = 0.143), grade (*p* = 0.619), and radiotherapy (*p* = 0.857) whereas more lymph nodes were dissected in lobectomy group (*p* < 0.001).

**Table 1:** The baseline characteristics of enrolled patients stratified by different treatment schemes.

Characteristics	Lobectomy (*n* = 106)	Sublober resection (*n* = 70)	Non-surgical (*n* = 315)	*p*
**Age**				<0.001
<65	38 (35.8)	18 (25.7)	50 (15.9)	
≥65	68 (64.2)	52 (74.3)	265 (84.1)	
**Gender**				0.852
Male	47 (44.3)	31 (44.3)	148 (47.0)	
Female	59 (55.7)	39 (55.7)	167 (53.0)	
**Race**				0.069
White	99 (93.4)	66 (94.3)	274 (87.0)	
Black	4 (3.8)	4 (5.7)	29 (9.2)	
Others	3 (2.8)	0 (0)	11 (3.5)	
Unknown	0 (0)	0 (0)	1 (0.3)	
**Laterality**				0.048
Left	42 (39.6)	20 (28.6)	140 (44.4)	
Right	64 (60.4)	50 (71.4)	175 (55.6)	
**Tumor location**				0.067
Upper lobe	64 (60.4)	53 (75.7)	187 (59.4)	
Middle lobe	10 (9.4)	2 (2.9)	24 (7.6)	
Lower lobe	32 (30.2)	15 (21.4)	104 (33.0)	
**Pathological T stage**				0.003
1a	69 (65.1)	51 (72.9)	167 (53.0)	
1b	37 (34.9)	19 (27.1)	148 (47.0)	
**T size (mm)**	18.2 ± 6.2	16.8 ± 6.8	20.6 ± 6.4	<0.001
**N number**				<0.001
0	4 (3.8)	33 (47.1)	306 (97.1)	
1 to 3	12 (11.3)	13 (18.6)	1 (0.3)	
4 or more	85 (80.2)	22 (31.4)	4 (1.3)	
Unknown	5 (4.7)	2 (2.9)	4 (1.3)	
**Grade**				<0.001
Well	3 (2.8)	0 (0)	1 (0.3)	
Moderate	2 (1.9)	1 (1.4)	2 (0.6)	
Poor	35 (33.0)	22 (31.4)	39 (12.4)	
Undifferentiated	33 (31.1)	28 (40.0)	52 (16.5)	
Unknown	33 (31.1)	19 (27.1)	221 (70.2)	
**Radiotherapy**				<0.001
Yes	23 (21.7)	16 (22.9)	215 (68.3)	
No	83 (78.3)	54 (77.1)	100 (31.7)	

**FIGURE 1:**
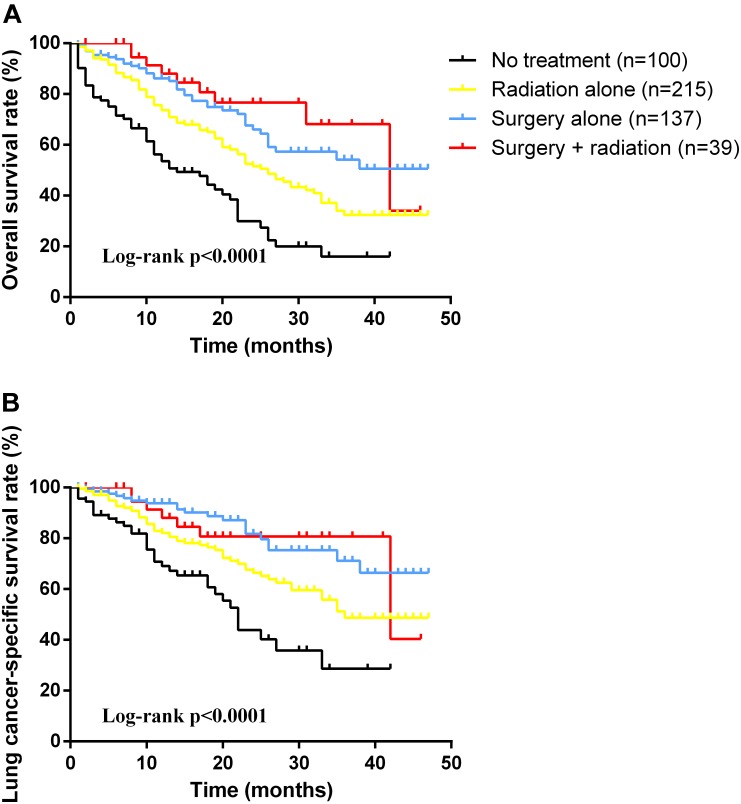
Kaplan-Meier survival analyses of overall survival **(A)** and lung cancer-specific survival **(B)** based on different treatment schemes in patients with stage IA small cell lung cancer.

**FIGURE 2:**
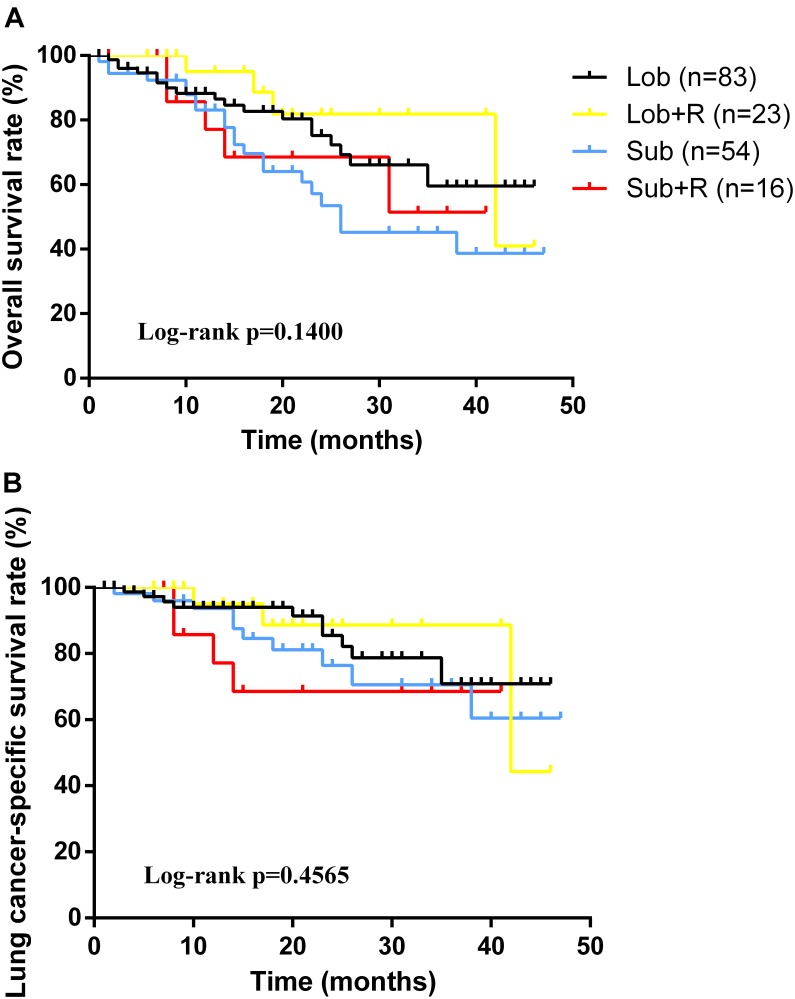
Kaplan-Meier survival analyses of overall survival **(A)** and lung cancer-specific survival **(B)** based on different treatment schemes in stage IA small cell lung cancer patients who underwent surgery.

**FIGURE 3:**
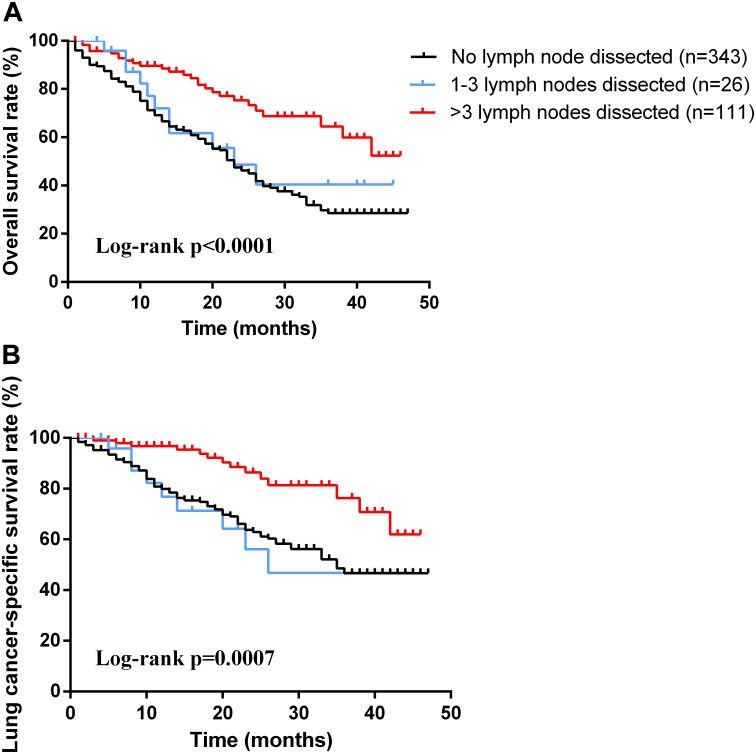
Kaplan-Meier survival analyses of overall survival **(A)** and lung cancer-specific survival **(B)** based on different numbers of lymph node dissection in patients with stage IA small cell lung cancer.

As for the survival, there were significant differences among the groups with different treatment schemes in OS (log-rank *p* < 0.0001) and LCSS (log-rank *p* < 0.0001) (Figure 1). Besides, patients who received surgery plus postoperative radiotherapy experienced the longest survival time (Figure 1). In subgroup analyses, there was no difference among the groups based on different surgical procedures both in OS (log-rank *p* = 0.14) and LCSS (log-rank *p* = 0.4565). However, survival in patients with lobectomy was better than those with sublober resection in trend (Figure 2). Moreover, postoperative radiotherapy would help improving the survival both in lobectomy group and sublober resection group (Figure 2). More lymph nodes dissected would lead to better survival both in OS (log-rank *p* < 0.0001) and LCSS (log-rank *p* = 0.0007) (Figure 3).

Univariable analysis revealed that age, laterality, tumor location, N number, and grade were significant predictors of OS while age, laterality, tumor location, and N number were significant predictors of LCSS (Table 2). Furthermore, age, laterality, tumor location, and N number were still significant predictors of OS, whereas age, tumor location, and N number were significant predictors of LCSS in multivariable analysis (Table 3).

**Table 2:** Univariable analyses for OS and LCSS.

	OS			LCSS		
Variable	HR	95% CI	*P*	HR	95% CI	*P*
Age, years	1.750	1.205–2.543	0.003	1.734	1.072–2.804	0.025
Gender	0.796	0.604–1.048	0.103	0.842	0.589–1.202	0.344
Race	0.989	0.711–1.375	0.947	1.148	0.782–1.684	0.481
Laterality	0.733	0.556–0.966	0.027	0.695	0.487–0.994	0.046
Tumor location	0.823	0.702–0.964	0.016	0.706	0.566–0.879	0.002
Pathological T stage	1.069	0.810–1.412	0.635	1.022	0.713–1.467	0.905
T size	1.005	0.985–1.027	0.611	1.009	0.982–1.037	0.498
N number	0.654	0.542–0.789	<0.001	0.681	0.538–0.862	0.001
Grade	1.268	1.072–1.499	0.006	1.191	0.965–1.469	0.104
Radiation	0.805	0.611–1.060	0.122	0.918	0.642–1.311	0.637

*OS, overall survival; LCSS, lung cancer specific survival; HR, hazard ratio; CI, confidence interval.*

**Table 3:** Multivariable analyses for OS and LCSS.

	OS			LCSS		
Variables	HR	95% CI	*P*	HR	95% CI	*P*
Age, years	1.564	1.072–2.282	0.020	1.630	1.005–2.644	0.048
Laterality	0.756	0.573–0.996	0.047	0.712	0.498–1.018	0.062
Tumor location	0.800	0.683–0.938	0.006	0.695	0.559–0.865	0.001
N number	0.699	0.576–0.848	<0.001	0.703	0.554–0.890	0.004
Grade	1.139	0.956–1.357	0.146			

*OS, overall survival; LCSS, lung cancer specific survival; HR, hazard ratio; CI, confidence interval.*

## Discussion

Lung cancer maintains the leading cause of death from cancer around the world. The treatment of SCLC, with the characteristics of rapid growth and early metastasis, is still an intractable problem. Although some researchers verified the effect of surgery on early-staged SCLC (stage I) (Ahmed et al., 2017), no previous studies focused on the equivalency of lobectomy versus sublober resection among stage IA SCLC patients. In the current study of stage IA SCLC patients, we analyzed the prognosis (OS and LCSS) among groups based on different treatment schemes. Our findings revealed that surgery is an important part of multidisciplinary treatment for stage IA SCLC patients and sublober resection is not inferior to lobectomy for the specific patients. Sublober resection could preserve more lung parenchyma and have reduced overall mortality when compared to lobectomy, considering that the clinicopathological data are unavailable in SEER database, whether sublober resection could be recommended for stage IA SCLC patients still need further study.

As NCCN Guidelines suggested, chemotherapy acts as an essential part of appropriate regimens for all SCLC patients, especially for those with surgical resection, no matter limited-stage or extensive-stage disease (Ettinger et al., 2018). Radiotherapy is also recommended for concurrent use with chemotherapy, but the optimal dose and schedule of radiotherapy has not reached a consensus. In our study, patients who received radiotherapy alone could acquire better survival than those without treatment in both OS (log-rank *p* < 0.0001) and LCSS (log-rank *p* = 0.0016). Moreover, surgery plus radiotherapy could achieve the best prognosis. Ahmed et al. (Ahmed et al., 2017) analyzed stage I SCLC patients based on the SEER database, and they also found patients with surgery plus radiation owned the longest survival, which is in concordance with our findings.

As for stage IA SCLC patients without mediastinal lymph nodes involved, surgery should be considered (Schneider et al., 2011). In early days, surgery alone could not be identified as a significant benefit for patients with limited-stage SCLC (Fox and Scadding, 1973; Osterlind et al., 1985). Recently, most of the retrospective studies regarding surgery in early-staged SCLC patients have revealed improved survival with surgical resection (James et al., 2010; Combs et al., 2015). Weksler et al. (2012) identified 3566 stage I or II SCLC patients in SEER database from 1988 to 2007, and the findings showed patients who underwent surgical resection had better outcomes when compared with those without surgery (median, 34.0 months versus 16.0 months, *p* < 0.001). Moreover, they also found patients who underwent lobectomy or pneumonectomy experienced significant longer survival than those underwent wedge resection (median, 39.0 months versus 28.0 months, *p* < 0.001). Similar findings were vertified by another study (Ahmed et al., 2017). Although many researchers in favor of lobectomy for early-staged SCLC patients due to the aggressive characteristics of the tumor, and they thought lobectomy plus lymph node dissection could achieve complete resection, we did not observe any survival differences between lobectomy group and sublober resection group in our study. The reason would be: (1) the tumor of stage IA SCLC was small and harbors relatively weaker invasiveness; (2) the number of patients with sublober resection in the set was relatively small, which would cause some bias.

Adequate lymph node dissection also made sense for overall survival. The dissected number of lymph nodes was identified as a significant predictor of OS and LCSS. The removal of four or more lymph nodes yielded important long-term benefit in survival for stage IA SCLC patients. Besides, adequate lymph node dissection is helpful in the determination of pathological tumor staging, choice of therapy and prediction of prognosis.

Our results also showed that elderly patients (65 years or more) were less likely to receive surgical resection (*p* < 0.001). The probable reasons may be the higher incidence of comorbidities and poorer lung function (Jazieh et al., 2002). Similarly, McCann et al. (2005) suggested that the lower surgical rate of surgical resection for older patients because of lower performance status and concurrent comorbidities.

The limitations of the study are as follows. First, it was a retrospective study and the nature of retrospective analysis may cause selection bias. Second, despite SEER database is a population-based data, many clinicopathological variables are unavailable, such as lung function, clinical tumor stage, comorbidities, adequacy of resection margin, and neoadjuvant or adjuvant chemotherapy. Consequently, the effect of chemotherapy could not be evaluated and the heterogeneity of enrolled patients would exist. However, as pathological stage IA SCLC, when compared with advanced SCLC, the number of stage IA SCLC patients who received preoperative radiochemotherapy is much smaller. Thus, the deficiency of preoperative radiochemotherapy data has limited influence on our conclusions. Third, the number of patients who underwent surgery were relatively small, and thus we could not further investigate the equivalency of wedge resection versus segmentectomy in stage IA SCLC patients. Prospective studies are required to further confirm the role of different surgical procedures in stage IA SCLC patients.

In summary, our findings revealed that surgery is an important component of multidisciplinary treatment for stage IA SCLC patients and sublober resection is not inferior to lobectomy for the specific patients. But these findings still need to be verified by further prospective researches.

## Author Contributions

CC conceived and designed the study, and provided the administrative support. CG, ZH, and CD provided the study materials or patients, and collected and assembled the data. CG, YW, and YR analyzed and interpreted the data. All authors wrote the manuscript and approved the final version of the manuscript.

## Conflict of Interest Statement

The authors declare that the research was conducted in the absence of any commercial or financial relationships that could be construed as a potential conflict of interest.
